# Mediastinal bronchogenic cyst resected in Kinshasa, Democratic Republic of Congo. A case report

**DOI:** 10.1016/j.ijscr.2022.107775

**Published:** 2022-11-15

**Authors:** Alphonse Nzomvuama Ndonga N'sungu, Théodore Junior Sakaji Manyka, Joël Noutakdie Tochie

**Affiliations:** a Department of Thoracic and Cardiovascular Surgery, Kinshasa University Hospital, Faculty of Medecine, Democratic Republic of the Congo; b Department of Surgery, Centre Medical de Kinshasa, Democratic Republic of the Congo; c Department of Emergency, Anesthesiology and Critical Care Medecine, Laquintinie Hospital, Douala, Cameroon

**Keywords:** Bronchogenic cyst, DR Congo, Mediastinum, Surgery

## Abstract

**Introduction:**

Bronchogenic cyst (BC) is an uncommon, benign congenital mediastinal tumor. Its progressive course is often asymptomatic, making the diagnosis difficult or fortuitous, unless non-specific respiratory signs appear, such as persistent cough or respiratory discomfort. We herein report the first documented case in the Democratic Republic of Congo (DRC).

**Presentation of case:**

A 63-year-old man, with no prior medical or surgical history, consulted for a persistent dry cough, unresponsive to usual medical treatment. A chest CT scan revealed a cystic mediastinal mass close to both esophagus and aorta. This mass was completely resected through a left posterior thoracotomy. Histological examination confirmed the diagnosis of bronchogenic cyst. The postoperative course was uneventful. The cough was completely resolved.

**Discussion:**

BC is the most common cystic mass of the mediastinum. The case of BC that we report is the first documented and published in the DRC. Diagnosis is often fortuitous, unless clinical signs appear, usually cough, dyspnea, and in some cases fever, recurrent respiratory infections. The surgical resection of the BC must be complete to avoid recurrence, to prevent and treat possible complications. Surgery removes any diagnostic doubt by allowing histological examination of the cystic mass.

**Conclusion:**

We reported our first resection of a BC. The preoperative diagnosis is often incidental. Complete resection of any BC allows histological diagnosis, treatment or prevention of complications.

## Highlights

• Bronchogenic cyst, a rare and asymptomatic tumor.
• Thoracic CT scan is the benchmark exam.
• Treatment: surgical resection

## 1 Introduction

Bronchogenic cyst (BC) is an uncommon congenital benign tumor mainly found in the mediastinum. The estimated incidence of BC ranges from 10 % to 15 % of all primary mediastinal masses [1]. The growth of a BC is usually asymptomatic, making its diagnosis difficult and sometimes fortuitous. Symptomatic patients have non-specific signs, mainly related to mechanical compression by the cystic mass. Respiratory signs such as cough, dyspnea, recurrent respiratory infections, and even signs of cardiac tamponade have been described [2], [3]. Surgical resection of the BC is the only effective and efficient treatment [4], [5].

We herein report the first documented surgical management of a BC in an adult in the Democratic Republic of Congo, following the 2020 SCARE criteria [6].

## 2 Presentation of case

**Fig. 1:**
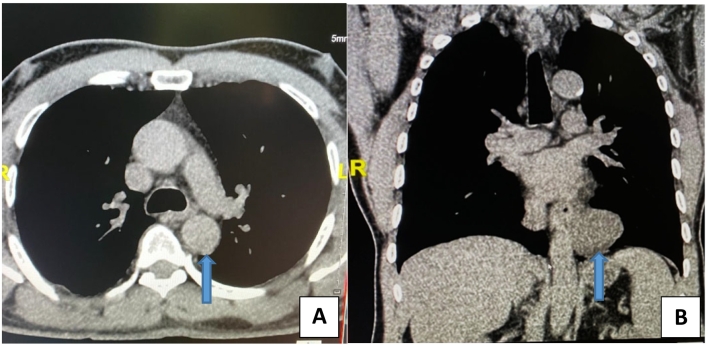
A rounded cystic mass (arrow) of the posterior mediastinum, axial (A) and coronal (B) chest CT scan.

A 63-year-old man with no significant medical or surgical history consulted for a dry and persistent, feverless cough since more than three months. The cough was unresponsive to anti-cough medication and antibiotics. This patient had no history of active smoking. Screening for active tuberculosis was negative. All other biological tests were normal. Neither was the chest X-ray contributive. However, the thoracic CT scan showed a 50 mm thin-walled cystic mass located in the lower middle mediastinum, close to the esophagus and the thoracic aorta (Fig. 1A and B).

**Fig. 2:**
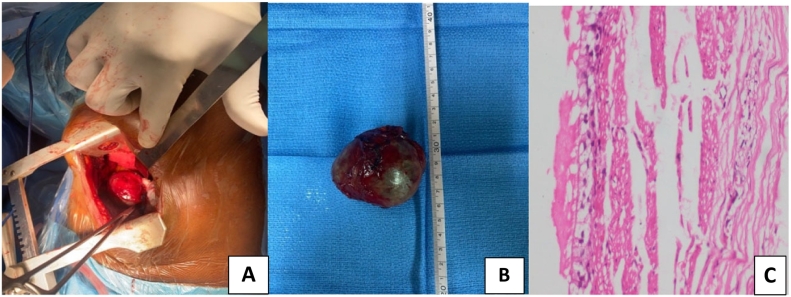
(A): An operative view of the mass through thoracotomy; (B): 6 × 5 cm smooth and thin-walled bronchogenic cyst; (C). a lined wall of columnar or cuboidal ciliated epithelium reminiscent of a normal bronchus.

A complete resection of the cyst was performed through a posterior thoracotomy at the sixth intercostal space (Fig. 2A and B). Histopathological analysis showed a cyst wall lined by respiratory epithelium with inflammatory changes and no evidence of cytonuclear atypia. (Fig. 2C).

The postoperative evolution was simple, mainly characterized by cough resolution. The patient was discharged from hospital on the 9th postoperative day. At the 7th postoperative month, he was doing well and had resumed work.

## 3 Discussion

Bronchogenic cyst (BC) arises from the ventral side of the primitive foregut. Following the growth of the respiratory bud between the 20th and 40th day of gestation, the respiratory and digestive tracts cleave. A BC can develop at this time. An early formed BC is thought to be preferentially located in the mediastinum, near the esophagus, trachea, carina and bronchi [7], [8].

Although rare, BC is the most common cystic mass of the mediastinum [1]. Indeed, most BCs are found in the mediastinum and, to a lesser extent, in the lungs. However, other, even rarer localizations, including cardiac, diaphragmatic and retroperitoneal, have also been described [2], [3].

The case of BC that we report is, to our knowledge, the first documented and published in our country. We performed a complete resection of the BC without any adverse events and the postoperative course was uneventful.

Our challenge is less about the technique than about the diagnosis. Access to imaging technology is not equally available throughout our country. CT scanning is mostly confined to urban cities and the cost of the exam is high for most of our patients. Therefore, there may be cases of undiagnosed BC. Furthermore, it is also possible that other surgical teams, without reporting their experience, have resected BC in our country, which is a true subcontinent in terms of size, with at least 90 million inhabitants.

BC remains asymptomatic in more than 70 % of cases and up to 90 % in mediastinal forms [1]. The only complaint of our patient was cough. Despite the closeness of the esophagus, the patient did not report any dysphagia. The persisting cough led first to the assessment of tuberculosis because of its high prevalence in our setting. However, BC and tuberculosis, although very rare, have been described [9].

Most cases of BC in the literature concern children, even neonates. BC is thus described as cases of life threatening cardio-respiratory distress, which is a consequence of mechanical pressure on the surrounding organs: tamponade by cardiac chambers compression or collapsing of the tracheobronchial tree facilitated by its compliance in infancy [2], [3], [10].

As we report here, BC is also seen in adults. Diagnosis is often fortuitous, unless clinical signs appear, usually cough, dyspnea, and in some cases fever, recurrent respiratory infections. The chest radiograph has a poor diagnostic value. The thoracic CT scan is the benchmark exam, providing useful preoperative information such as shape and size of the cyst, location and relationship to adjacent structures [3].

Finding a circumferential mass on the thoracic CT scan, usually with liquid content, is a strong argument for the diagnosis of BC. However, in many cases, due to its mucoprotein-rich content, BC may have a density close to that of solid masses, rather than a low density close to water as would be expected from a cyst [9].

Only histological exam can provide diagnostic confirmation showing a lined wall of columnar or cuboidal ciliated epithelium, reminiscent of a normal bronchus with occasional hyaline cartilage, smooth muscle, elastic tissue, and mucous glands [3], [11]. In fact, differential diagnosis may be discussed with various congenital or acquired pathologies, including cystic teratoma, bronchopulmonary sequestration, esophageal cysts, pulmonary abscess, hydatid cyst, infected bullae, lobar emphysema, etc.

We completely resected this patient's BC through a thoracotomy, free of adverse intraoperative events despite the close contact to the esophagus and the vicinity of the descending aorta. Nowadays, thorascopic surgery has become the first-line technique. Our limited resources did not allow its use. Thoracotomy is still indicated by some authors for large, strongly adherent or complicated cysts [7], [12].

The surgical resection of the BC must be complete to avoid recurrence and, above all, to prevent possible complications. Surgery removes any diagnostic doubt by allowing histological examination of the cystic mass [4]. Surgical indication is not controversial for symptomatic cysts.

There is no evidence, however, that resection of asymptomatic BCs is necessary. Data on long-term follow-up of never operated BCs are missing. The risk of complications associated with their evolution justifies their preventive resection. Indeed, most BCs ultimately become either symptomatic or complicated. Some cases of malignant involvement have been described [10], [12].

## 4 Conclusion

BC is usually a silent disease. Diagnosis is often fortuitous, unless some signs of complications occur. We reported our first resection of a BC. The postoperative course has been uneventful. The challenge for us, as elsewhere, remains the diagnosis. Systematic and complete resection of any BC ensures the establishment of its histological diagnosis and the treatment or the prevention of its potential complications.

## Funding

N/a.

## Ethical approval

N/a.

## Consent

Written informed consent was obtained from the patient for publication of this case report and accompanying images. A copy of the written consent is available for review by the Editor-in-Chief of this journal on request.

## Registration of research studies

N/a.

## Guarantor

Alphonse Nzomvuama.

## CRediT authorship contribution statement

All authors contributed toward data analysis, drafting, and revising the paper. They gave final approval of the version to be published, and agree to be accountable for all aspects of the work.

## Declaration of competing interest

No competing of interest in this study.
